# Association of Myopia With Risk of Incident Metabolic Syndrome: Findings From the UK Biobank Study Cohort of 91,591 Participants

**DOI:** 10.3389/fmed.2022.872013

**Published:** 2022-05-16

**Authors:** Yanxian Chen, Zhuoting Zhu, Wei Wang, Xianwen Shang, Mingguang He, Jinying Li

**Affiliations:** ^1^Department of Ophthalmology, Peking University Shenzhen Hospital, Shenzhen Peking University-The Hong Kong University of Science and Technology Medical Center, Shenzhen, China; ^2^Department of Ophthalmology, Guangdong Academy of Medical Sciences, Guangdong Provincial People's Hospital, Guangzhou, China; ^3^State Key Laboratory of Ophthalmology, Zhongshan Ophthalmic Center, Sun Yat-sen University, Guangzhou, China; ^4^Centre for Eye Research Australia, Ophthalmology, Department of Surgery, University of Melbourne, Melbourne, VIC, Australia

**Keywords:** myopia, metabolic syndrome, older population, Western lifestyle, cohort study

## Abstract

**Purpose:**

To investigate the association between myopia and risk of metabolic syndrome (MetS) in a prospective cohort from the UK Biobank Study.

**Methods:**

Volunteers (aged 40 years and above) free of baseline MetS and cataract included from the UK Biobank Study, a prospective follow-up cohort. Myopia was defined using uncycloplegic autorefraction, self-report-myopia, and medical records for refractive error at baseline. MetS as well as components of MetS were diagnosed based on health records, blood biochemistry, and questionnaires. Questionnaires determined the status of smoking, drinking, physical activity and dietary supplements, as well as ethnicity and education.

**Results:**

A total of 91,591 participants were available in the analysis, with a mean age of 55.37 ± 8.07 years at baseline and a median follow-up years of 11.16 years. The proportion of myopia was 49.7%, and a total of 937 (1.0%) participants were identified as having incident MetS (0.09/100 person years). Subjects with myopia were more likely to have MetS compared with non-myopic subjects (0.82 vs. 0.21%, Log-rank test *P* < 0.001). Mopes had greater risk of incident MetS (Hazard ratio [HR] = 4.19, 95% confidence interval [CI] 3.57–4.93, *P* < 0.001) adjusting for baseline age, gender, education and ethnicity. After further controlling for lifestyle factors (smoking, drinking, physical activity, and fish oil supplement) or baseline metabolic disorders, the risk of incident MetS were 3.88- and 4.06-fold greater in myopic subjects than those without myopia, respectively (*P* < 0.001 for both models). The severity of myopia was not significantly correlated to incident MetS in multivariate-adjusted models.

**Conclusions:**

An increased risk of incident MetS among the elderly is associated with myopia, but not the degree of myopia. These findings highlighted the need of prevention of MetS among older adults with myopia.

## Introduction

In recent decades, myopia has become a major ocular condition leading to visual impairment with a rapidly increasing prevalence, especially in areas with rapid urbanization, including South Korea (1), Taiwan (2), and mainland China (3). It has been estimated that the global prevalence of myopia will be as high as 50% by 2050 (4). Environmental factors predominantly contribute to the current rise in prevalent myopia, including intensive near work and less time outdoors (5, 6), which are common patterns of activities in modern lifestyle, and may continue to impact behavioral aspects of living (7, 8).

Metabolic syndrome (MetS) is a metabolic disorder involving central obesity, high blood pressure, hyperglycemia and dyslipidemia, closely relating to Western obesogenic diet and lifestyle (9). It has been long postulated that there may be common pathogenetic basis for metabolic disorder and myopia due to the wide acceptance of Western lifestyle over the last few decades (10–12). But the association between myopia and the individual components of MetS have been reported with some inconsistency. For example, in the Handan Eye Study, diabetes mellitus was a risk factor for myopia in the elderly (13), but the Blue Mountains Eye study (14) and the Beaver Dam Eye Study (15) showed no significant association between myopia and diabetes. Elevated systolic blood pressure was observed in highly myopic patients (16) and myopic children (17), while hypertension was not associated with myopia in the Tanjong Pagar Survey (18). There have also been conflicting associations between obesity (or increased body mass index) and myopia (19–21). Heretofore the association between MetS and myopia has not fully identified in existing studies.

In this study, we aimed to investigate the association of myopia and different degree of myopia with incident MetS in a large-scale prospective cohort from the UK Biobank Study.

## Materials and Methods

### Population

The UK Biobank is a large-scale prospective cohort study of health and wellbeing enrolling more than 500,000 participants aged 40–69 years at baseline recruitment between 2006 and 2010 throughout the United Kingdom. Detailed methodology for the UK Biobank Study has been described previously (22). In brief, about 9.2 million subjects aged 40–69 years in 22 assessment centers were invited to participate, and data from 502,505 people were collected (response rate 5.5%). The baseline assessment visit including comprehensive questionnaires, physical and functional measures and collection of biological samples. Ophthalmic assessments were conducted in 6 assessment centers. Non-cycloplegic autorefraction was performed with the Tomey rc-5000 Auto Refkeratometer (Tomey, Nagoya, Japan) and up to 10 measurements were taken for each eye. A detailed procedure can be seen elsewhere (23). Follow-up visits were performed in 2012–2013, 2014, and 2019.

Ethical approval was obtained from the National Information Governance Board for Health and Social Care and the NHS North West Multicenter Research Ethics Committee. Written informed consent was obtained from all participants.

### Definitions

Myopia was defined using non-cycloplegic autorefraction and questionnaires data. Subjects with spherical equivalent (sphere + 1/2 cylinder) < −0.50D in either eye were considered to have myopia. For subjects without autorefraction data, hospital health record, self-reported eye disease and reason for glasses in questionnaires were used to identify status of refractive error. High myopia was defined as an SE < -6.0D, or with self-reported high myopia in questionnaires. Participants who underwent refractive surgery for myopia were also included as myopes. We excluded subjects who had cataract, amblyopia or strabismus diagnosis from hospital record and previous cataract surgery.

In the current study, MetS was defined using the International Diabetes Federation (IDF) criteria (24): (1) Waist circumference > 94 cm in Europid men and >80 cm in Europid women [for Chinese and South Asian ethnicity was >90 cm for men and >80 cm for women, adapted from Kendal DM et tal. (25)]; (2) Blood pressure > 130/85 mmHg or treatment of previously diagnosed hypertenstion; (3) Triglyceride > 1.7 mmol/l or specific treatment for lipid abnormality; (3) HDL-C <1.0 mmol/l in men, <1.3 mmol/l in women or specific treatment for lipid abnormality; (4) Fasting plasma glucose > 5.6 mmol/l or previously diagnosed type 2 diabetes. Diagnosis of MetS was considered when the individual met criterion (1) plus any two of the rest disorders. The level of triglyceride, HDL-C and glucose were available in blood biochemistry data. History of treatment for hypertension, diabetes, or lipid abnormality were extracted from questionnaires.

Incident MetS in follow-up period were identified as the proportion of subjects who had no MetS at baseline and who subsequently developed MetS during the follow-up period. Follow-up time was defined as the duration between the date of baseline visit and the date of following status which was the earliest: incident MetS, lost to follow-up, or death.

Potential confounders associating with MetS or myopia were also identified in the analysis. Information of smoking status (previous/current and never), drinking status (previous/current and never) at baseline, educational level (collage/university/NVQ and others), ethnicity (white and non-white) were obtained from questionnaires. Level of physical activity was identified as low, moderate and high using International Physical Activity Questionnaire (IPAQ), and further categorized to above moderate/vigorous/walking recommendation and not, according to Metabolic Equivalent Task scores based on IPAQ guidelines (26). Information of dietary supplements were collected from questionnaires and recorded as fish oil supplement and others. Hypertension was defined as a systolic blood pressure >130 mmHg or a diastolic blood pressure >80 mmHg, or a self-reported hypertension, or taking antihypertensive drugs. Diabetes mellitus was defined as a glycosylated hemoglobin level of >6.5%, or a self-reported diabetes mellitus, or taking medication for diabetes mellitus.

### Statistical Analysis

We used *t* tests to determine differences in baseline age between participants with myopia and without myopia, and between those with mild/moderate myopia and with high myopia. Pearson's χ^2^ test were used for comparison of categorical variables. Survival distribution was compared using Log-rank test, and Cox proportional hazards regression models was used to assess the associations between covariates and incident MetS. All analysis were performed using Stata (version 16.0, StataCorp).

## Results

**Figure 1:**
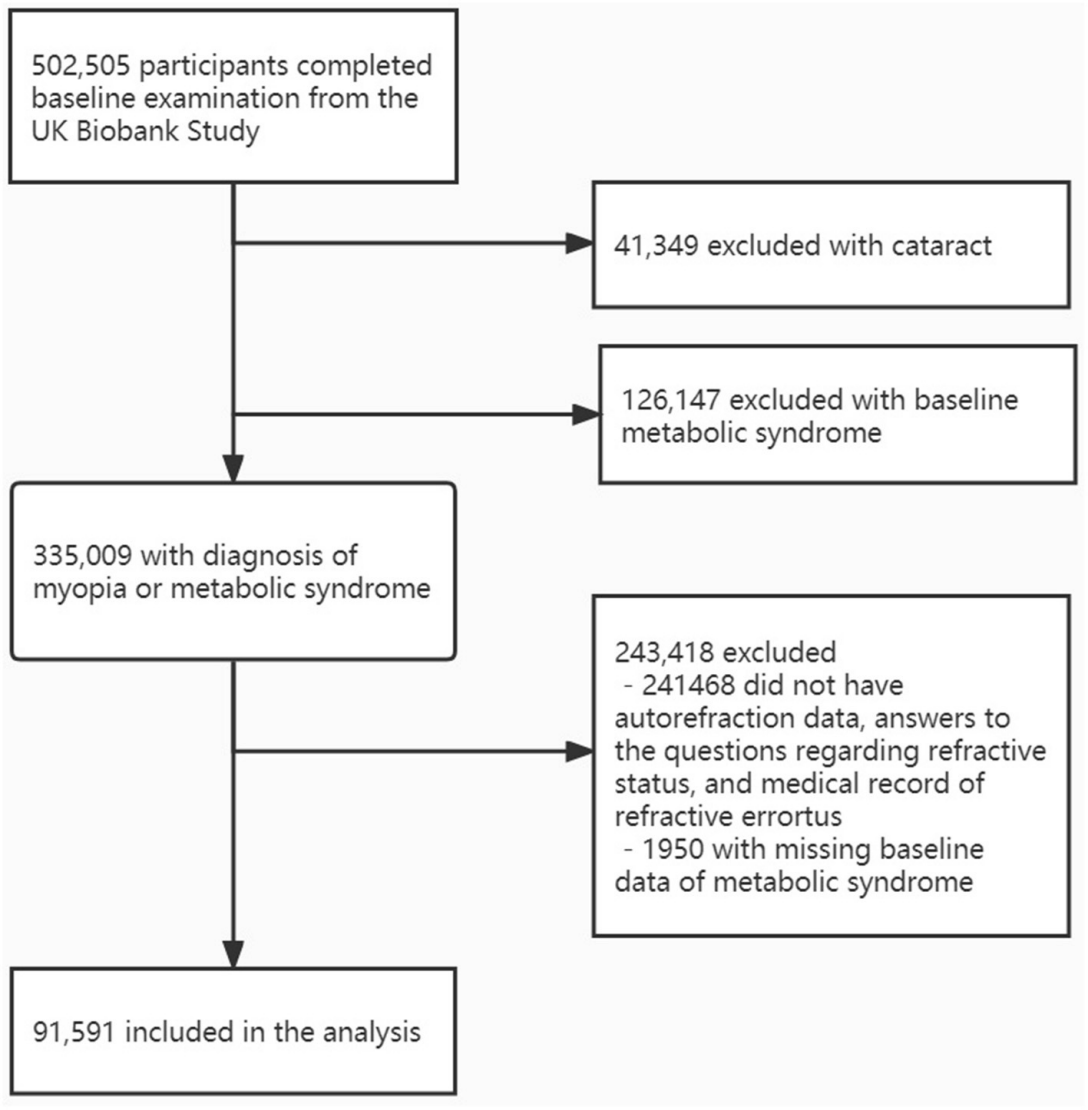
Flowchart depicting inclusion of participants in this study.

Among the 502,505 participants in the baseline UK Biobank Study, myopia diagnosis was identified in 65,875 subjects: 44,373 with available autorefraction data, 1,361 with history of refractive surgery for myopia, 2,753 with self-reported myopia and 17,388 with hospital medical record of myopia diagnosis. As showed in Figure 1, after excluding subjects with cataract (*n* = 41,349), baseline MetS (*n* = 126,147) and missing data on refraction status (without autorefraction data, answers to the questions regarding refractive status, and medical records of refractive error, *n* = 241,468) or baseline data of MetS (*n* = 1,950), a total of 91,591 subjects were available in the analysis, among which 45,487 were myopic (49.7%) and 3,650 were highly myopic (4.0%). Subjects included in our analysis were younger, had more females, more college graduates, and less white people compared with the 410,914 subjects who were excluded from the analysis (Supplementary Table 1).

Baseline characteristics between myopes and non-myopes were summarized in Table 1. Compared with non-myopic group, myopic group had a significantly younger baseline age (54.32 ± 7.82 years vs. 56.40 ± 8.19 years, *P* < 0.001), more females (55.1 vs. 55.9%, *P* = 0.026), and a higher proportion of subjects with college degree (52.6 vs. 38.9%), more white people (92.0 vs. 90.0%, *P* < 0.001), more central obesitive (42.1 vs. 35.9%, *P* < 0.001), more hypertensive (48.3 vs. 47.5%, *P* = 0.022), less smokers (37.8 vs. 45.2%, *P* < 0.001), more drinkers (96.4 vs. 95.2%, *P* < 0.001), less physically active (82.2 vs. 84.9%, *P* < 0.001), less fish oil supplement (29.3 vs. 30.6%, *P* < 0.001). The distributions of gender, diabetes mellitus, high triglyceride and low HDL-C showed no significant difference between myopic and non-myopic group.

**Table 1:** Baseline characteristics of participants with and without myopia.

**Variable**	**No myopia** **(*n* = 46,104)**	**Myopia** **(*n* = 45,487)**	**Total (*n* = 91,591)**	***P***
Baseline age, year, mean (sd)	56.40 (8.19)	54.32 (7.82)	55.37 (8.07)	<0.001
Female, *n* (%)	25,759 (55.9%)	25,082 (55.1%)	50,841 (55.5%)	0.026
College/university degree, *n* (%)	17,921 (38.9%)	23,915 (52.6%)	41,836 (45.7%)	<0.001
Ethnicity, *n* (%)				<0.001
White	41,491 (90.0%)	41,851 (92.0%)	83,342 (91.0%)	
Others	4,613 (10.0%)	3,636 (8.0%)	8,249 (9.0%)	
Central obesity, *n* (%)	19,393 (42.1%)	16,337 (35.9%)	35,730 (39.0%)	<0.001
Hypertension, *n* (%)	21,904 (47.5%)	21,954 (48.3%)	43,858 (47.9%)	0.022
Diabetes Mellitus, *n* (%)	659 (1.4%)	621 (1.4%)	1,280 (1.4%)	0.408
High triglyceride, *n* (%)	13,420 (29.1%)	13,053 (28.7%)	26,473 (28.9%)	0.169
Low HDL-C, *n* (%)	2,974 (7.7%)	3,053 (8.0%)	6,027 (7.9%)	0.060
Smoker, *n* (%)	20,705 (45.2%)	17,123 (37.8%)	37,828 (41.5%)	<0.001
Drinker, *n* (%)	43,697 (95.2%)	43,754 (96.4%)	87,451 (95.8%)	<0.001
PA above moderate/vigorous/walking recommendation, *n* (%)	31,868 (84.9%)	31,629 (82.2%)	63,497 (83.5%)	<0.001
Fish oil supplement, *n* (%)	14,087 (30.6%)	13,189 (29.3%)	27,276 (30.0%)	<0.001

*PA, physical activity*.

In subjects with high myopia, the proportion of female was higher (58.5 vs. 55.1%, *P* < 0.001), educational level was higher (college graduates 59.6 vs. 49.8%, *P* < 0.001), proportion of central obesity was smaller (34.5 vs. 38.2%, *P* < 0.001), high triglyceride was lower (21.9 vs. 25.2%, *P* < 0.001) and number of smokers was less (33.0 vs. 39.6%, *P* < 0.001) compared with subjects with mild/moderate myopia (Supplementary Table 2).

There were 937 participants (1.0%, 0.09/100 person years) having incident MetS during follow-up period. The median (IQR) follow-up post baseline enrollment was 11.16 (0.55) years. Table 2 demonstrates baseline characteristics of participants with and without incident MetS. Subjects with incident MetS tended to be older (57.18 ± 7.09 years vs. 55.34 ± 8.08 years, *P* < 0.001), have less females (44.3 vs. 55.6%, *P* < 0.001), and more white people (97.0 vs. 90.9%, *P* < 0.001). The proportion of myopes in subjects with incident MetS was significantly higher (79.7 vs. 49.4%, *P* < 0.001) than in those without incident MetS, while the degree of myopia was similar between the two groups. The percentage of baseline central obesity, hypertension, diabetes mellitus, high triglyceride and low HDL-C were significantly higher in subjects with incident MetS than in those without (*P* < 0.001 for all), and the level of physical activity was lower (77.6 vs. 83.6%, *P* <0.001), but the number of smokers and drinkers did not differ significantly between the two groups.

**Table 2:** Baseline characteristics of participants with and without incident metabolic syndrome.

	**No incident MetS** **(*n* = 90,654)**	**Incident MetS** **(*n* = 937)**	***P***
Baseline age, year, mean (sd)	55.34 (8.08)	57.18 (7.09)	<0.001
Female, *n* (%)	50,426 (55.6%)	415 (44.3%)	<0.001
College/university degree, *n* (%)	41,380 (45.7%)	456 (48.7%)	0.065
Ethnicity, *n* (%)			<0.001
White	82,433 (90.9%)	909 (97.0%)	
Others	8,221 (9.1%)	28 (2.9%)	
Myopia, *n* (%)	44,740 (49.4%)	747 (79.7%)	<0.001
High myopia, *n* (%)	3,860 (12.8%)	21 (14.9%)	0.451
Central obesity, *n* (%)	35,190 (38.8%)	540 (57.6%)	<0.001
Hypertension, *n* (%)	43,044 (47.5%)	814 (86.9%)	<0.001
Diabetes Mellitus, *n* (%)	1,228 (1.4%)	52 (5.6%)	<0.001
High triglyceride, *n* (%)	25,609 (28.3%)	864 (92.2%)	<0.001
Low HDL, *n* (%)	5,927 (7.8%)	100 (14.1%)	<0.001
Smoker, *n* (%)	37,436 (41.5%)	392 (41.9%)	0.795
Drinker, *n* (%)	86,544 (95.8%)	907 (96.8%)	0.114
PA above moderate/vigorous/walking recommendation, *n* (%)	62,895 (83.6%)	602 (77.6%)	<0.001
Fish oil supplement, *n* (%)	26,982 (29.9%)	294 (32.7%)	0.066

*MetS, metabolic syndrome; PA, physical activity*.

**Figure 2:**
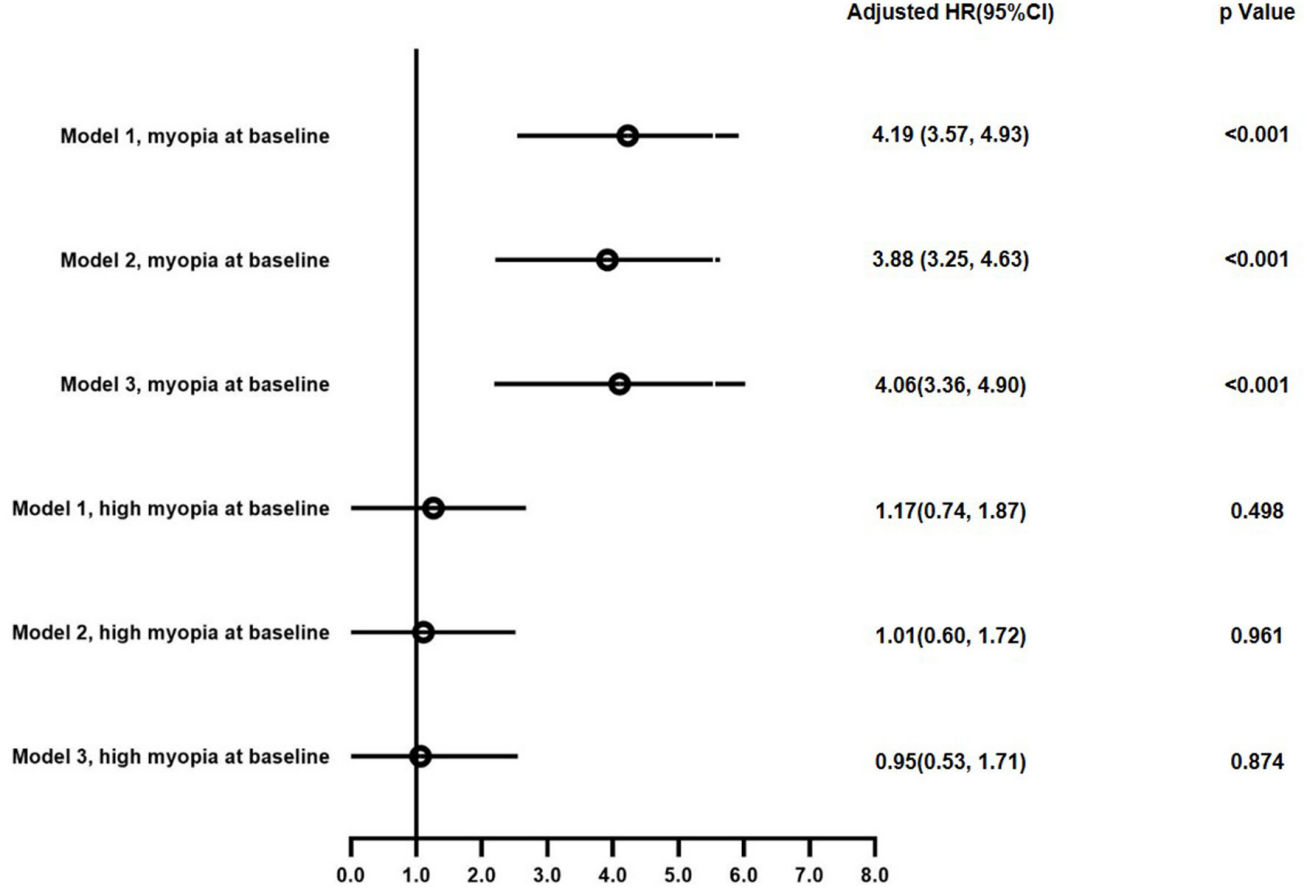
Hazard ratio with 95% confidence interval of myopia and degree of myopia for incident metabolic syndrome in multivariate Cox proportional-hazard model.

The incidence of MetS in myopes (0.82%, 0.14/100 person years) were significantly higher than those without baseline myopia (0.21%, 0.03/100 person years, Log-rank test *P* < 0.001), while incident MetS in different degree of myopia was not significantly different (0.13% [0.04/100 person years] in mild/moderate myopia vs. 0.02% [0.05/100 person years] in high myopia, Log-rank test *P* = 0.1315). We performed three different models to assess the contribution of myopia and myopia degree to incident MetS: Model 1 adjusted for baseline age, gender, ethnicity and education; Model 2 adjusted for variables in Model 1 plus smoking, drinking, physical activity level and fish oil supplement; Model 3 adjusted for variables in Model 1 plus baseline central obesity, hypertension, low HDL-C and high triglyceride. The coefficients with 95% confidence intervals (CIs) of each individual covariate in Cox proportional hazards regression model for incident MetS were listed in Supplementary Table 3. In summary, baseline age, baseline myopia, baseline central obesity, baseline hypertension, baseline diabetes, baseline high triglyceride and baseline low HDL-C level were independently associated with incident MetS. As seen in Figure 2, myopic subjects had a 4.19-fold higher risk of developing MetS (95%CI 3.57–4.93) in Model 1, and a 3.88-fold risk (95%CI 3.25–4.63) in Model 2. After further adjusting for baseline metabolic disorders, myopes still had a 3-times higher risk of future MetS (Hazard ratio 4.06, 95%CI 3.36–4.90) than non-myopes. However, high myopia at baseline was not a significant risk factor of incident MetS when adjusting for age, gender, education, ethnicity, lifestyle factors, or baseline metabolic disorders.

Sensitivity analysis was conducted within different subgroups of participants. As shown in Supplementary Figure 1, after excluding participants with college degree or above, or non-white ethnicity, or obesity/hypertension/smoker/low level of physical activity at baseline, the results were comparable to the main analysis, verifying the robustness of our findings.

## Discussion

In this large community-based follow-up study, being myopic at baseline was a significant risk factor of incident MetS later in life, and the association remained significant after adjusting for confounders. However, the severity of myopia was not independently associated with future risk of MetS.

To our best knowledge, the current analysis is the first study reporting the association between myopia and incident MetS. In consistent with other studies, incident MetS was strongly associated with baseline metabolic disorders, including obesity (27, 28), higher systolic blood pressure (28) and low HDL-C (29). Nevertheless, we found that myopia remained to be a significant predictor for incident MetS after controlling all the baseline metabolic disorders. Diabetes mellitus is a common metabolic abnormality related to myopia, mostly attribute to the influence of glucose level on lens power (30, 31). However, the prevalence of diabetes mellitus was only 1.4% at baseline in the current study. Therefore, we confirmed that myopia is associated with risk of developing MetS as an independent risk factor, not as a result of change in lens power due to metabolic disorders.

The insignificant association between degree of myopia and incident MetS was also observed in the current analysis. The proportion of high myopia in this study was 4.0%, similar to a 2–5% prevalence of high myopia in older population from population-based researches, where high myopia was considered to be more genetic in etiology (32–35). We can speculated that severe myopia is not correlated to incident MetS due to the weak connection with environmental factors. However, this possibility may need to be validated in a cohort consisted of congenital and acquired high myopia due to the lack of differentiation from types of myopia herein.

Several potential mechanisms may explain the association between myopia and the risk of MetS. Firstly, myopia and MetS share common environmental risk factors. Decreased outdoor activity has been well-recognized as a key determinant of myopia development (5, 36) and teenagers and young adults with myopia tend to spend less time on physical activity and more time sedentary or use of screen-based devices (8, 12, 37). Our results also suggested that adults with myopia tend to have a lower level of moderate/vigorous physical activity. Meanwhile, being more physically active significantly reduces risk for metabolic syndrome (38), which is in consistent with the current analysis. It is plausible that physical activity patterns in myopes influences the rate of MetS development later in life. Secondly, there might be interrelation in the pathogenesis between myopia and MetS. Vascular inflammation is commonly seen in pathophysiology of obesity (39, 40). diabetes mellitus (41, 42) as well as in myopia progression (3, 43). In addition, insulin has been found to promote myopia development both *in vitro* (44) and *in vivo* studies (45, 46). Polymorphism of insulin-like growth factor-1 (IGF-1), a downstream production of insulin, also is associated with severe myopia (47).

As the first study investigating the association between myopia and incident MetS, the implications of our findings are far reaching. From a socio-economic perspective, tremendous amounts of resources have been spent on prevention and mitigation of metabolic disorders (48–50). The association between myopia and incident MetS identified in our study indicates the burden on public health will be far more huge when the young people with higher prevalence of myopia grow older. From the perspective regarding service for individual patients as part of a shift toward “precision medicine,” active procedures are warranted in the future to reduce the risk of incident MetS in the elderly with myopia, including accessibility and uptake of regular medical checkup, as well as adapted instructions and exercise therapy for myopes.

Strengths of our study include the large-scale cohort from whom detailed health records identifying MetS and refractive errors were collected, and a detailed assessment of associations between incident MetS and potential risk factors. The main flaw of this study is the lack of axial length data, limiting further investigation of the association of MetS with axial myopia and lens-induced myopia. Future studies with a more comprehensive eye examination might be needed to explore underlying mechanism of the established association in this study. Another potential limitation is that we may not exclude all the confounders especially for the factors related to lifestyle, including sleep duration, time spent on outdoor activities or near work.

## Conclusions

In summary, we found that myopic subjects were at an increased risk of incident MetS controlling for baseline confounders, but the severity of myopia showed little influence on future development of MetS. The findings suggest the significance of prevention of MetS in older adults with myopia.

## Data Availability Statement

The data analyzed in this study is subject to the following licenses/restrictions: UK Biobank data are available only for applicants. Requests to access these datasets should be directed to https://www.ukbiobank.ac.uk.

## Ethics Statement

The studies involving human participants were reviewed and approved by the National Information Governance Board for Health and Social Care and the NHS North West Multicenter Research Ethics Committee. The patients/participants provided their written informed consent to participate in this study.

## Author Contributions

YC and JL devised the project. MH and XS assisted with project developing. ZZ and WW conducted data processing and analysis. YC wrote the manuscript. JL and MH reviewed and revised the manuscript. All authors contributed to the article and approved the submitted version.

## Conflict of Interest

The authors declare that the research was conducted in the absence of any commercial or financial relationships that could be construed as a potential conflict of interest.

## Publisher's Note

All claims expressed in this article are solely those of the authors and do not necessarily represent those of their affiliated organizations, or those of the publisher, the editors and the reviewers. Any product that may be evaluated in this article, or claim that may be made by its manufacturer, is not guaranteed or endorsed by the publisher.
