# Supplementary material for: Association of Myopia With Risk of Incident Metabolic Syndrome: Findings From the UK Biobank Study Cohort of 91,591 Participants
Source: Front Med (Lausanne). 2022 May 16;9:872013. doi: 10.3389/fmed.2022.872013 (PMC9150791; doi:10.3389/fmed.2022.872013)

**Supplementary**

Table S1. Baseline characteristics in included and excluded subjects

|  | **Excluded (n=410914)** | **Included (n=91591 )** | **P value** |
| --- | --- | --- | --- |
| Baseline age, year, mean (sd) | 56.15(8.04) | 55.4(8.07) | <0.001 |
| Female, n (%) | 198909 (53.8%) | 50841(55.5%) | <0.001 |
| Education, n(%) |  |  | <0.001 |
| College | 138435(37.5%) | 41836(45.7%) |  |
| Others | 231130(62.5%) | 49755(54.2%) |  |
| Ethnicity, n(%) |  |  | <0.001 |
| White | 351484(95.1%) | 83342(91.0%) |  |
| Others | 18081(4.9%) | 8249(9.0%) |  |

Table S2. Baseline characteristics of subjects with mild/moderate myopia and with high myopia

|  | **Mild/moderate myopia (n=24999)** | **High myopia (n=3650 )** | **P value** |
| --- | --- | --- | --- |
| Baseline age, year, mean (sd) | 54.45(7.96) | 54.20(7.93) | 0.0632 |
| Female, n (%) | 14604 (55.1%) | 2269(58.5%) | <0.001 |
| College/university degree, n(%) | 13200(49.8%) | 2312(59.6%) | <0.001 |
| White, n(%) | 23805(89.9%) | 3448(88.8%) | 0.050 |
| Central obesity, n(%) | 10109(38.2%) | 1340(34.5%) | <0.001 |
| Hypertension, n(%) | 12125(45.8%) | 1814(46.7%) | 0.259 |
| Diabetes Mellitus, n(%) | 411(1.6%) | 50(1.3%) | 0.210 |
| High triglyceride, n(%) | 7386(27.9%) | 964(24.8%) | <0.001 |
| Low HDL-C, n(%) | 1699(7.6%) | 274(8.5%) | 0.100 |
| Smoker, ,n(%) | 10454(39.6%) | 1277(33.0%) | <0.001 |
| Drinker, n(%) | 25341(95.9%) | 3717(96.0%) | 0.943 |
| Above moderate physical activity, n(%) | 18556(83.1%) | 2756(82.4%) | 0.364 |
| Fish oil supplement, n(%) | 7572(28.6%) | 1064(27.4%) | 0.128 |

Figure S1. Hazard ratios and 95% confidence interval in the sensitivity analysis using different subgroups of participants.


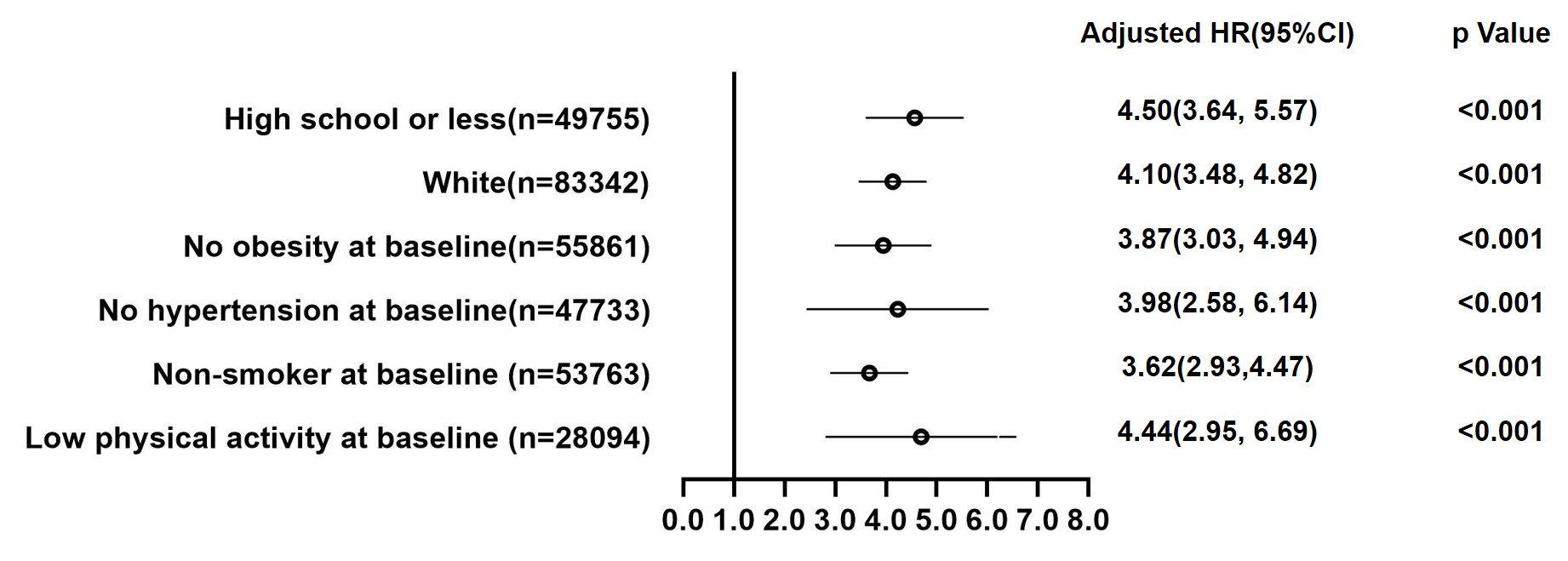

Supplement: Supplementary file 1 [file Data_Sheet_1.docx]
